# Notopterol Suppresses IL-17-Induced Proliferation and Invasion of A549 Lung Adenocarcinoma Cells via Modulation of STAT3, NF-κB, and AP-1 Activation

**DOI:** 10.3390/ijms242015057

**Published:** 2023-10-11

**Authors:** Sirinada Inthanon, Pornngarm Dejkriengkraikul, Supachai Yodkeeree

**Affiliations:** 1 Department of Biochemistry, Faculty of Medicine, Chiang Mai University, Chiang Mai 50200, Thailand; sirinada_i@cmu.ac.th (S.I.); pornngarm.d@cmu.ac.th (P.D.); 2 Anticarcinogenesis and Apoptosis Research Cluster, Faculty of Medicine, Chiang Mai University, Chiang Mai 50200, Thailand

**Keywords:** notopterol, interleukine-17, A549 cells, proliferation, invasion, MAPKs, STAT3, AP-1, NF-κB

## Abstract

Interleukine-17 is a proinflammatory cytokine that promotes lung cancer growth and progression though the activation of the STAT3, NF-κB, and AP-1 signaling pathways. Therefore, blocking the IL-17-induced oncogenic pathway is a new strategy for the treatment of lung cancer. Notopterol, a furanocoumarin, has demonstrated anti-tumor effects in several types of tumors. However, its molecular function in relation to the IL-17-induced proliferation and invasion of A549 lung adenocarcinoma cells remains unknown. Here, notopterol exhibited an inhibitory effect on IL-17-promoted A549 cell proliferation and induced G0/G1 cell cycle arrest. Western blot analysis revealed that notopterol inhibited the expression of cell-cycle-regulatory proteins, including cyclin D1, cyclin E, CDK4, and E2F. Moreover, notopterol blocked IL-17-induced A549 cell migration and invasion by regulating the epithelial–mesenchymal transition (EMT) and reducing the expression of extracellular degradation enzymes. At the molecular level, notopterol treatment significantly down-regulated the IL-17-activated phosphorylation of Akt, JNK, ERK1/2, and STAT3, leading to a reduced level of transcriptional activity of NF-κB and AP-1. Collectively, our results suggest that notopterol blocks IL-17-induced A549 cell proliferation and invasion through the suppression of the MAPK, Akt, STAT3, AP-1, and NF-κB signaling pathways, as well as modulating EMT.

## 1. Introduction

Lung cancer is widely recognized as the leading cause of cancer-related morbidity and mortality. Among the various types of lung cancer, non-small-cell lung cancer (NSCLC) accounts for approximately 80% of all cases. The five-year survival rate for NSCLC is notoriously low, with only 25–30% of patients with metastatic disease surviving for more than three months [1,2,3]. NSCLC itself can be further categorized into several different types, with the most common including squamous cell carcinoma, large cell carcinoma, and adenocarcinoma. Although several anti-cancer strategies, such as surgery, chemotherapy, and radiation therapy, are used to treat NSCLC, there is an urgent need for effective strategies to reduce the high mortality rates. Traditional chemotherapy shows limited efficacy due to a lack of precision and usually exhibits serious adverse effects. Immunotherapy is currently being applied in NSCLC. It has shown substantive clinical activity in metastatic lung cancer and is approved for first or subsequent lines of therapy. However, only 20% of NSCLC patients show significant clinical benefit due to the development of resistance [4]. Recent studies have shed light on the connection between NSCLC progression and metastasis and inflammation, specifically through the elevation of proinflammatory cytokine levels [5,6,7]. Inflammation has been found to promote tumor progression and development through various mechanisms, with interleukins playing a critical role in shaping the tumor microenvironment. Therefore, targeting inflammation can represent a new potential approach for the treatment of lung cancer.

Interleukin-17 (IL-17) is one of the proinflammatory cytokines that have been reported to induce NSCLC proliferation, angiogenesis, invasion, and immune tolerance [8,9]. Data obtained from clinical studies have revealed a significant correlation between the expression of IL-17 in tumor tissue and serum samples from NSCLC patients and their clinical stage and overall survival rate [10,11]. IL-17 promotes cancer progression by activating multiple signaling pathways, including JAK/STAT3, MAPK, PI3K/Akt, and NF-κB [9,12]. The activation of these complex signaling pathways leads to the induction of tumorigenic properties in cancer cells, including the upregulation of proliferation (cyclin D1), invasive (MMP-9, MT1-MMP, uPA, and uPAR), and inflammatory (IL-6 and TNF-α) proteins [13,14,15]. Furthermore, the IL-17-induced epithelial–mesenchymal transition (EMT) promotes metastasis by regulating the NF-κB and MAPK signaling pathways, as reported in previous studies [16,17,18]. The inhibition of IL-17 has been shown to suppress metastasis and improve sensitivity to both chemotherapy and radiation therapy in preclinical cancer models [7]. Therefore, efficient agents capable of blocking IL-17-mediated signaling pathways in tumors may provide a novel approach to controlling tumor growth and metastasis.

Notopterol, a type of furanocoumarin, is a bioactive compound isolated from the rhizomes and roots of *Notopterygium incisum* using chloroform. In recent years, the biological activities of notopterol have been reported, suggesting potential anti-inflammatory, analgesic, and anti-hypertensive properties [19,20]. Interestingly, notopterol has shown anticancer activity in various cancer cell types, including leukemic, breast, and hepatic cancer cells. Its anti-tumor mechanisms are very diverse, including inducing G0/G1 phase arrest in HL-60 promyelocytic leukemia cells by regulating cell cycle proteins, promoting apoptosis by increasing apoptotic proteins, and suppressing hepatocellular carcinoma cell viability by inhibiting JAK2 activation and enhancing oxidative stress [21,22,23]. Furthermore, notopterol reduces the production of inflammatory cytokines by inhibiting the JAK-STAT3 and STAT3/NF-κB signaling pathways [24]. However, the effect of notopterol on IL-17-induced NSCLC proliferation and metastasis has not yet been elucidated.

The A549 human lung adenocarcinoma cell line has been widely employed to investigate various aspects of lung cancer biology, including cell proliferation, apoptosis, angiogenesis, metastasis, and the mechanisms of action of various anti-cancer agents. Furthermore, studies have reported that IL-17 induces invasion and proliferation in A549 cells [7,13]. This underscores the value of the A549 cell line as a valuable tool for examining the impact of notopterol on IL-17-promoted tumor progression. In the current study, A549 cells were treated with IL-17 and notopterol. After treatment, we assessed cell viability, proliferation, and invasion. Additionally, we explored the mechanism of notopterol on IL-17-induced cell proliferation by examining the expression of cell cycle and cell proliferation proteins. Moreover, we investigated the expression of proteins associated with metastasis. Furthermore, we determined the effects of notopterol on the MAPK, Akt, STAT3, NF-κB, and AP-1 signaling pathways in IL-17-induced A549 cells.

## 2. Results

### 2.1. Effect of Notopterol on the Cytotoxicity of A549 Lung Adenocarcinoma Cells

**Figure 1:**
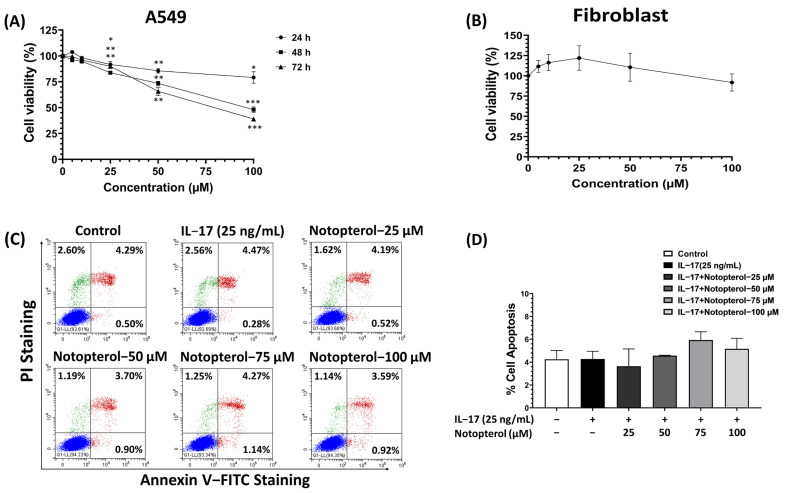
Effect of notopterol on cell viability in A549 and human skin fibroblast cells. (**A**) A549 cells were treated with notopterol (0–100 μM) for 24, 48, and 72 h and cell viability was detected via SRB assay. (**B**) Human dermal skin fibroblasts were incubated with notopterol for 48 h and cell viability was determined via SRB assay. (**C**) Apoptotic cells were detected via annexin V and PI staining in combination. Treatment of A549 cells with notopterol (0–100 μM) and IL-17 (25 ng/mL) for 48 h was detected using annexin V and PI staining and analyzed via flow cytometry. (**D**) Graphical representation of percentage of apoptosis cells. Data are presented as mean ± S.D. values of three independent experiments. * *p* < 0.05, ** *p* < 0.01, *** *p* < 0.001 compared to the control.

The cytotoxicity of notopterol was examined via SRB assay. As shown in Figure 1A, the treatment of A549 cells with various concentrations (0–100 μM) of notopterol for 24, 48, and 72 h significantly reduced the cell viability of A459 cells with dose and time dependence. In particular, the inhibition concentrations at 50% notopterol at 48 and 72 h were 90.6 µM and 80 μM, respectively. In contrast, notopterol at 100 μM did not affect the viability of normal human fibroblast cells (Figure 1B). Next, we investigated whether the cytotoxicity effect of notopterol on A549 cells was associated with apoptosis by using an Annexin VI-PI staining assay. The result indicated that treating the cells with notopterol at 100 μM revealed no significant increase in the apoptotic population when compared to the control (Figure 1C,D).

### 2.2. Notopterol Inhibits IL-17 Stimulation of A549 Cell Proliferation

**Figure 2:**
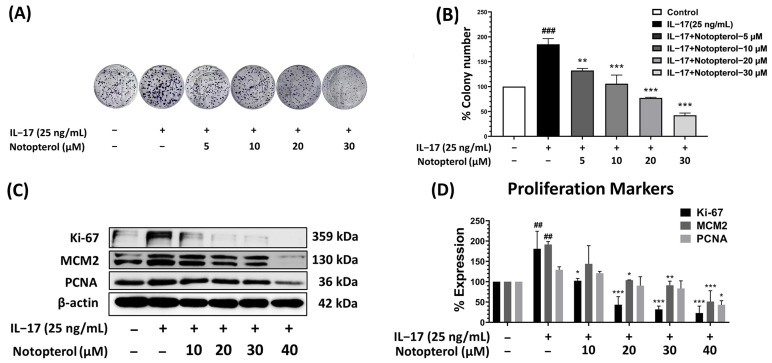
Anti-proliferative effect of notopterol on IL-17-induced A549 cells. The cells were exposed to IL-17 (25 ng/mL) and notopterol (0–30 μM) for 10 days. (**A**) The proliferation of A549 cells was assessed using a colony formation assay. (**B**) Representative bar graph from colony assay. (**C**) The effects of notopterol on the expression of proteins involved in proliferation in IL-17-induced A549 cells were detected using Western blot analysis. (**D**) Quantitative representation of band density for the expression levels of proliferative protein markers (Ki-67, MCM2, and PCNA) normalized to β-actin. Data are presented as mean ± S.D. values of three independent experiments. ## *p* < 0.01, ### *p* < 0.001 compared to control group; * *p* < 0.05, ** *p* < 0.01, *** *p* < 0.001 compared to IL-17 (25 ng/mL).

To assess the potential reduction of IL-17-promoted proliferation in A549 cells by notopterol, we subjected the cells to combination treatments involving various concentrations of notopterol and IL-17 (25 ng/mL). Cell proliferation was then determined using a colony formation assay. The results revealed that notopterol markedly decreased IL-17-induced colony formation in a dose-dependent manner (Figure 2A,B). We next assessed the effect of notopterol on the expression of proliferation proteins. The results presented in Figure 2C,D indicate that the induction of Ki-67, MCM-2, and PCNA by IL-17 was reduced by notopterol in a dose-dependent manner. 

### 2.3. Notopterol-Induced Cell Cycle Arrest

**Figure 3:**
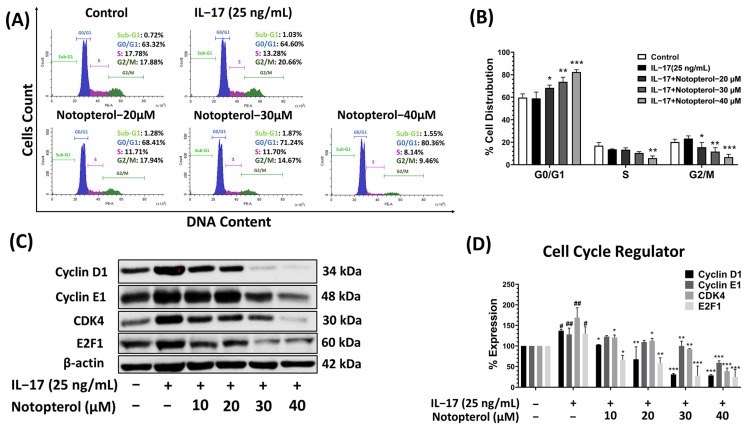
Notopterol affects cell cycle distribution in A549 cells. The cells were treated with notopterol (0–40 μM) and IL-17 (25 ng/mL) for 24 h. (**A**) The cell cycle was determined via PI staining and analyzed via flow cytometry to detect cell cycle distribution. (**B**) The data presented in a bar graph. (**C**) The expression of cell-cycle-regulatory proteins (cyclin D1, cyclin E, CDK4, and E2F) was detected via Western blot analysis, and (**D**) densitometric and statistical analysis of protein quantification data are presented as a histogram. Data are presented as mean ± S.D. values of three independent experiments. # *p* < 0.05, ## *p* < 0.01, compared to control group; * *p* < 0.05, ** *p* < 0.01, *** *p* < 0.001 compared to IL-17 (25 ng/mL).

To investigate the relationship between the anti-proliferative activity of notopterol and cell cycle arrest, A549 cells were treated with both IL-17 and notopterol. Subsequently, the cells were stained with PI, and cell cycle progression was examined using flow cytometry. The treatment of cells with notopterol at 30 and 40 μM significantly increased the cell population in the G0/G1 phase, while decreasing the number of cells in the S and G2/M phases compared to treatment with IL-17 alone (Figure 3A,B). Next, the effect of notopterol on the expression of G0/G1-regulatory proteins was evaluated using Western blot analysis. As shown in Figure 3C,D, the induction of Cyclin D1, Cyclin E, CDK4, and E2F by IL-17 was reduced by notopterol in a dose-dependent manner. Taken together, these results indicate that notopterol can reduce the IL-17-induced enhancement of A549 cell proliferation by inducing cell cycle arrest.

### 2.4. Notopterol Suppressed IL-17-Induced A549 Cell Invasion and Migration

**Figure 4:**
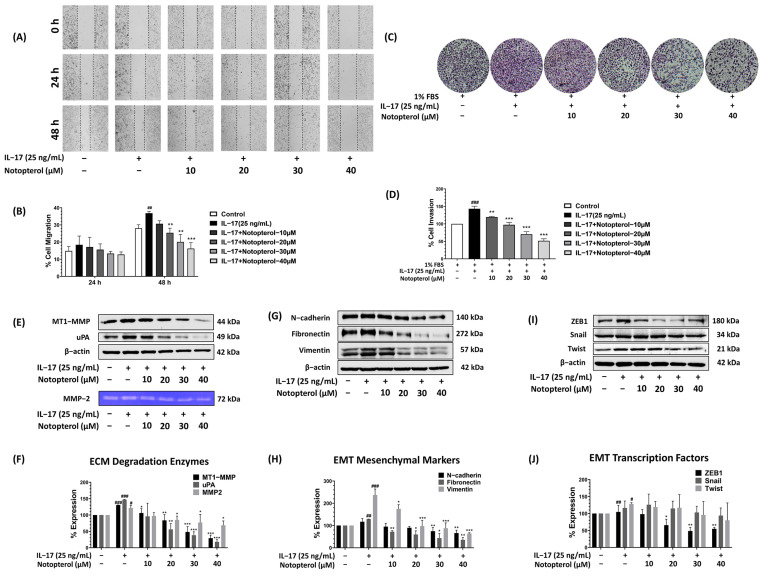
Notopterol inhibits IL-17-induced A549 cell invasion and migration. (**A**) Cell migration in A549 cells was assessed after exposure to IL-17 (25 ng/mL) and varying concentrations of notopterol (0–40 μM) for 24 and 48 h using a wound healing assay. (**B**) The bar graph represents the percentage of cell migration, quantified as the closure of the scratch area. (**C**) Boyden chamber assay was performed to detect the effect of notopterol on IL-17-induced invasion of A549 cells, and (**D**) the percentage of cell invasion is presented in a bar graph. After co-treating the cells with notopterol and IL-17 for 24 h, (**E**) whole-cell extracts were prepared and analyzed via Western blot using antibodies against metastatic proteins (MT1-MMP and uPA), and the culture supernatant was used to analyze the level of MMP-2 via gelatin zymography. (**F**) The bar graph represents the expression of ECM degradation enzymes. (**G**) The expression level of EMT markers was analyzed via Western blot analysis, and (**H**) the histogram graph represents the band intensity of EMT marker proteins. (**I**) The level of EMT transcription factors, including ZEB-1, Snail, and Twist, were investigated via Western blot, and (**J**) the band intensity is presented in a bar graph. Data are presented as mean ± S.D. values of three independent experiments. # *p* < 0.05, ## *p* < 0.01, ### *p* < 0.001 compared to control group; * *p* < 0.05, ** *p* < 0.01, *** *p* < 0.001 compared to IL-17 (25 ng/mL).

IL-17 has been shown to promote the migration and invasion of lung cancer cells. Therefore, we evaluated the effect of notopterol on the IL-17-induced migration and invasion of A549 cells using wound healing and Boyden chamber invasion assays. As shown in Figure 4A,B, the wound healing capacity significantly increased in A549 cells after 48 h of IL-17 treatment compared to the control. However, in the presence of notopterol, the IL-17-induced migration of A549 cells was significantly inhibited. Furthermore, IL-17 also stimulated A549 cell invasion 1.5-fold, while notopterol suppressed this activity in a dose-dependent manner (Figure 4C,D). As IL-17 promotes cancer cell metastasis by upregulating invasive proteins, we investigated the effect of notopterol on the expression of uPA, MT1-MMP, and MMP-2. Western blot analysis revealed that notopterol significantly decreased the IL-17-induced expression of uPA and MT1-MMP. In addition, gelatin zymography indicated that notopterol reduced MMP-2 expression in a dose-dependent manner (Figure 4E,F). EMT is thought to be a key mechanism by which tumor cells facilitate their migration and invasion. The available evidence indicates that IL-17 induces EMT in lung cancer cells by promoting the expression of mesenchymal proteins and inhibiting epithelial proteins. Therefore, we determined the levels of EMT marker proteins, including N-cadherin, fibronectin, and vimentin, in A549 cells in the presence of notopterol. As shown in Figure 4G,H, the induction of N-cadherin, fibronectin, and vimentin by IL-17 was decreased by notopterol in a dose-dependent manner. Moreover, notopterol reduced the expression level of the EMT transcription factor ZEB1 but had no effect on Twist and Snail (Figure 4I,J). These results suggest that notopterol may exert its anti-migratory and anti-invasive effects on A549 cells by modulating ECM degradation enzymes and EMT proteins.

### 2.5. Effect of Notopterol on IL-17-Induced Oncogenic Signaling Pathways

**Figure 5:**
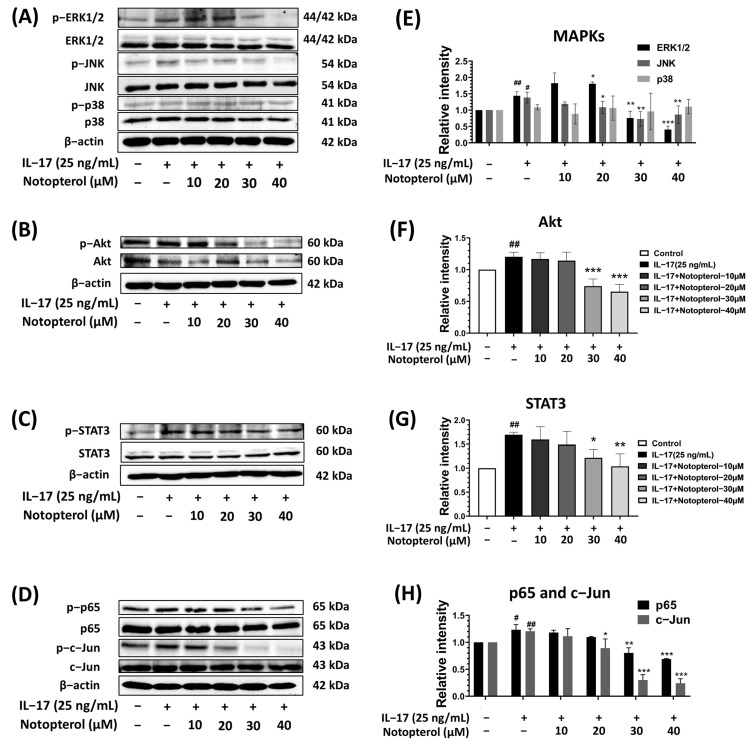
Effect of notopterol on IL-17-induced oncogenic signaling pathway. A549 cells were pretreated with notopterol (0–40 µM) and induced with IL-17 (25 ng/mL) for 1 h, and whole-cell extracts were subjected to Western blot analysis. The expression levels of phosphorylated and non-phosphorylated forms of oncogenic signaling pathways, including (**A**) MAPK, (**B**) Akt, (**C**) STAT3, (**D**) NF-κB (p65), and AP-1 (c-Jun), were determined via Western blot analysis. Band density quantification for (**E**) MAPKs, (**F**) Akt, (**G**) STAT3, (**H**) NF-κB, and AP-1 was performed using Image J (v1.410) and is presented in the histogram. Data are presented as mean ± S.D. values of three independent experiments. # *p* < 0.05, ## *p* < 0.01 compared to control group; * *p* < 0.05, ** *p* < 0.01, *** *p* < 0.001 compared to IL-17 (25 ng/mL).

Since the IL-17 activation of MAPKs and the Akt signaling pathway has been reported to be associated with proliferation and metastasis in lung cancer cells, we investigated the effect of notopterol on the IL-17-induced activation of Akt and MAPKs, including ERK1/2, p38, and JNK, using Western blot analysis. As shown in Figure 5A,E, notopterol inhibited the IL-17-induced phosphorylation of ERK1/2 and JNK in a dose-dependent manner, but it had no effect on the phosphorylation of p38. On the other hand, the treatment of A549 cells with notopterol significantly decreased IL-17-induced phosphorylation of the Akt signaling pathway (Figure 5B,F). In addition, it has been reported that IL-17 promotes lung cancer invasion and proliferation through the STAT3 pathway. Therefore, we investigated the effect of notopterol on IL-17-induced STAT3 activation using Western blot analysis. Notopterol treatment resulted in significant reductions in IL-17-induced STAT3 phosphorylation in a dose-dependent manner (Figure 5C,G). AP-1 and NF-κB transcription factors are involved in IL-17’s promotion of the progression of lung cancer. AP-1 and NF-κB are activated by the MAPK and Akt signaling pathways. To investigate whether notopterol affected IL-17-induced AP-1 and NF-κB activation, we determined the phosphorylation of c-Jun (AP-1) and p65 (NF-κB). As shown in Figure 5D,H, IL-17 enhanced the phosphorylation of c-Jun and p-65. Notopterol could inhibit the IL-17-indcued phosphorylation of AP-1 and NF-κB in a dose-dependent manner. These findings reveal that notopterol regulates A549 cell proliferation and invasion through multiple signaling pathways, including MAPK, Akt, STAT3, AP-1, and NF-κB.

## 3. Discussion

In recent years, an increasing number of reports have identified the important role of IL-17 in the development of various cancers, including gastric, lung, and breast cancer [25,26,27]. The levels of IL-17 are elevated in the tumor microenvironment and bloodstream in lung cancer, and this is associated with tumor progression [28,29]. Several studies have demonstrated that IL-17 can promote NSCLC proliferation, contributing to NSCLC growth and development. Therefore, targeting IL-17 could be a new potential therapeutic option against cancer. Notopterol is a furanocoumarin found in the root of *Notopterygium incisum*. It has displayed activity against many types of cancer by regulating the cell cycle, inducing apoptosis, and suppressing cancer stemness signaling [22,23]. Despite its various pharmacological activities, the molecular mechanism of notopterol in the context of IL-17-mediated tumor progression has not been investigated.

In this study, we found that notopterol significantly decreased IL-17-induced A549 cell proliferation but had no effect on cell apoptosis. The anti-cell-proliferation effect of notopterol was confirmed by investigating the expression levels of proliferative markers, including Ki-67, PCNA, and MCM2. Notopterol can suppress the upregulation of these proliferation markers in IL-17-induced A549 cells. The cell cycle determines cell proliferation and regulates the complex processes that govern cell growth and division. The signaling pathways affecting the cell cycle must be precisely regulated for cell cycle entry and progression. To date, numerous anticancer drugs have been demonstrated to arrest the cell cycle at specific phases [30,31]. Therefore, we investigated whether the inhibition of IL-17-induced A549 cell proliferation by notopterol is associated with cell cycle arrest. Here, we observed that notopterol induced the cell cycle arrest of A549 cells at the G0/G1 phase in a dose-dependent manner. The progression of cells from G0/G1 to the S phase is controlled by the activation of the E2F transcription factor and Cyclin-CDK complexes [32]. Upon growth stimulation, the expression of cyclin D1 is induced, forming an activating complex with CDK4/6. The cyclin D1-CDK4/6 complex phosphorylates retinoblastoma proteins, leading to their dissociation from E2F transcription factor. E2F initiates the transcription of genes involved in the cell cycle, including cyclin E, cyclin A, and CDC2, thereby promoting cell cycle progression from the G0/G1 phase to the S phase [33,34]. Previous observations have indicated that IL-17 promotes A549 cell proliferation through the overexpression of cyclin D1 [13]. Numerous studies have revealed that the inhibition of cyclin D1 and E2F induces cell cycle arrest at the G0/G1 phase [35,36,37]. To explore the mechanism by which notopterol induces cell cycle arrest in A549 cells at the G0/G1 phase, we assessed the expression levels of cell-cycle-regulatory proteins through Western blot analysis. The results showed that notopterol decreased the IL-17-induced expression of cyclin D1, E2F, cyclin E, and CDK4. These data are consistent with previous reports, which indicated that notopterol induced cell cycle arrest at the G0/G1 phase in human acute myeloid leukemia cells by inhibiting the expression of cyclin D, cyclin E, and CDK4 [22]. In summary, our results clearly demonstrate that notopterol modulates G1-phase regulatory proteins, resulting in the arrest of A549 cells.

Tumor metastasis is a major contributor to the death of cancer patients. The process of metastasis is a multistage mechanism, starting with the loss of intercellular connections, extracellular matrix (ECM) degradation, invasion of the basement membrane, penetration into the blood or lymphatic vessels, extravasation, and finally, regrowth at the metastatic site [38,39]. ECM degradation is one of the key steps in cancer cell metastasis. The main group of enzymes responsible for ECM degradation includes MMPs and uPA [40,41]. Therefore, interrupting ECM degradation can inhibit invasion and reduce the rate of tumor metastasis. Previous observations have indicated that IL-17 stimulates the expression of MMP-9 and MMP-2, promoting A549 cell invasion and migration [7]. Our results clearly demonstrate that notopterol suppresses the IL-17-induced invasion and migration of A549 cells. Moreover, notopterol reduces the levels of the IL-17-induced expression of invasive proteins, including MMP-2, MT1-MMP, and uPA, in A549 cells. Emerging evidence indicates that EMT is considered one of the most critical steps in cancer metastasis. EMT has been shown to correlate with disease progression and a worse prognosis in various cancer types, including lung adenocarcinoma [42,43]. Thus, inhibiting EMT progression may be beneficial for preventing cancer metastasis. In addition, it has been reported that IL-17 promotes the migration, invasion, and EMT process of lung cancer cells [44]. Therefore, we examined the effect of notopterol on the expression of EMT marker proteins such as N-cadherin, fibronectin, and vimentin, as well as the corresponding transcription factors, Snail, Twist, and ZEB-1, in IL-17-induced A549 cells. Here, we have shown that notopterol reduced the level of the IL-17-induced expression of mesenchymal markers, including N-cadherin, fibronectin, and vimentin, while the expression of the ZEB-1 transcription factor was suppressed by notopterol, but not Snail and Twist. These findings are consistent with another report indicating that notopterol suppresses cell invasion in cancer cells by modulating the EMT process [23]. Therefore, it is theorized that notopterol blocks IL-17-induced A549 cell invasion, at least partly through the modulation of ECM degradation enzymes and EMT marker expression levels.

It is widely accepted that IL-17 promotes cancer development and progression by modulating oncogenic kinase signaling pathways, including the MAPK, PI3K/Akt, and JAK/STAT3 pathways [7,9]. The activation of the MAPK and Akt signaling pathways has been implicated in cancer cell growth and metastasis as they are thought to upregulate the expression of proliferation and invasive proteins [45,46]. MAPKs are serine–threonine protein kinases composed of three well-defined subgroups, namely, ERK1/2, JNK1/2, and p38. Additionally, IL-17 stimulates cancer cells by utilizing the MAPK and Akt signaling pathways to promote cell proliferation and metastasis [47]. Therefore, experiments were performed to investigate whether notopterol regulates IL-17-induced activation of the MAPK and Akt signaling pathways. According to our results, notopterol prevented the IL-17-induced phosphorylation of Akt, JNK1/2, and ERK1/2. In contrast, notopterol did not affect the phosphorylation of p38. These findings are consistent with another report indicating that notopterol arrests osteoclastogenesis by inhibiting the MAPK signaling pathway [48]. According to the literature, the JAK/STAT3 pathway is one of the vital signaling pathways responsible for cancer cell proliferation and metastasis [49,50]. STAT3 is a transcription factor that is sequestered in an inactive form in the cytoplasm. The activation of STAT3 is predominantly regulated by upstream JAK kinases in various cancer types, including lung cancer. Activated STAT3 is translocated to the nucleus, where it promotes cell proliferation and cell cycle progression. STAT3 has been found to undergo phosphorylation by several pro-inflammatory cytokines, including IL-17 [51]. Accumulating evidence indicates that activation of the IL-17/JAK/STAT3 pathway plays a significant role in tumor metastasis and growth [52]. Numerous studies have demonstrated that suppression of the JAK/STAT3 pathway by inhibitors can inhibit cancer cell invasion and promote apoptosis [44,53]. Our study also showed that notopterol significantly inhibits the IL-17-induced phosphorylation of STAT3 in A549 cells. These observations are consistent with a previous study indicating that notopterol binds and targets the JAK/STAT3 pathway to ameliorate inflammation and arthritis [24]. This demonstrates the higher efficiency of notopterol in restraining the activation status of the MAPK, Akt, and STAT3 signaling pathways induced by IL-17. 

Transcription factors NF-κB and AP-1 have been extensively studied for their various roles in cancer development. NF-κB and AP-1 regulate the expression of multiple genes involved in tumor proliferation and metastasis, including those encoding cyclin D1, cyclin E, Myc, MMP-9, MT1-MMP, uPA, Snail, and ZEB1 [54,55]. Notably, the activation of the PI3K/Akt and MAPK pathways can transmit their signals to NF-κB and AP-1 in many cancer types [56]. The NF-κB and AP-1 signaling pathways are classic pathways that are activated by IL-17 [57]. Another report has indicated that IL-17 promotes cancer cell proliferation via the NF-κB signaling pathway [58]. In the current study, we also found that IL-17 induced the phosphorylation of NF-κB and AP-1. Moreover, the treatment of A549 cells with notopterol reduced NF-κB and AP-1 activity by inhibiting IL-17-induced p65 and c-Jun phosphorylation. These findings are consistent with those from previous studies, indicating that the suppression of NF-κB and AP-1 signaling can inhibit lung cancer cell proliferation and metastasis [59]. Based on the above-mentioned results, we suggest that notopterol could decrease the levels of proliferation- and metastasis-related proteins by suppressing NF-κB and AP-1 activation.

In summary, notopterol exerts a significant inhibitory effect on IL-17-induced A549 cell proliferation and invasion. Furthermore, notopterol treatment downregulates the MAPK, Akt, STAT3, NF-κB, and AP-1 signaling pathways, thereby modulating the expression of proliferation-, cell cycle-, ECM degradation-, and EMT-related proteins. This study has provided novel insights into the role of notopterol as a potential agent for targeting IL-17-promoted lung cancer cell progression, suggesting its potential in future lung cancer treatments. However, the present study has some limitations. This work initially investigated the functional role of notopterol in IL-17-induced lung cancer through in vitro experiments. In future research, a mouse model should be constructed to explore the influence of notopterol on the growth and metastasis of lung cancer in vivo. Additionally, notopterol exhibits limited solubility in culture media. Therefore, the preparation of notopterol-loaded nanoparticles before administration to the mouse may improve the solubility and bioavailability of notopterol.

## 4. Materials and Methods

### 4.1. Chemicals and Reagents

Dulbecco’s modified Eagle’s medium (DMEM), penicillin/streptomycin, and trypsin-EDTA were purchased from Gibco (Grand Island, NY, USA). Fetal bovine serum (FBS) was supplied by Hyclone (Logan, UT, USA). An FITC Annexin V kit was obtained from Elabscience Biotechnology (Houston, TX, USA). Antibodies specific to ERK1/2, p-c-Jun, p-p65 (p-NF-κB), p-Akt, Cyclin E, CDK4, N-cadherin, Vimentin, and β-actin were obtained from Cell Signaling Technology (Danvers, MA, USA), whereas antibodies specific to p-ERK, p38, p-p38, STAT3, p-STAT3, Akt, c-Jun, MCM2, PCNA, Cylin D1, E2F, Fibronectin, ZEB-1, MT1-MMP, uPA, and Claudin-1 were purchased from Abclonal (Woburn, MA, USA). Antibodies for the detection of Ki67, Snail, and p65(NF-κB) were purchased from Santa Cruz Biotechnology (Santa Cruz, CA, USA). Nitrocellulose membrane and ECL reagent were supplied by GE Healthcare (Little Chalfont, UK). Matrigel was purchased from Becton Dickinson (Bedford, MA, USA). Notopterol with purity of 98% was ordered from Chengdu Biopurify Phytochemicals Ltd. (Chengdu, China).

### 4.2. Cells and Cell Cultures

The A549 cells and human dermal fibroblasts were obtained from the American Type Culture Collection (Manassas, VA, USA). Both cell types were cultured in Dulbecco’s Modified Eagle’s Medium (DMEM) containing penicillin (100 U/mL), streptomycin (100 mg/mL), and fetal bovine serum (FBS) (10% *v*/*v*) at 37 °C in a 5% CO_2_ humidified incubator. For notopterol treatment, notopterol was dissolved in DMSO, and then, diluted with the culture medium, ensuring that the final concentration of DMSO was less than 0.5% (*v*/*v*).

### 4.3. Cell Viability Assay

The cytotoxicity of notopterol on A549 cells was determined using the sulforhodamine B (SRB) assay. A549 cells and fibroblast cells were plated at a density of 3.0 × 10^3^ cells/well in a 96-well plate and incubated at 37 °C in a 5% CO_2_ atmosphere overnight. They were then treated with various concentrations of notopterol (0–100 µM) for 24, 48, and 72 h. At the end of the treatment period, 100 µL of 10% (*w*/*v*) trichloroacetic acid (TCA) was added to fix the cells, followed by incubation at 4 °C for 1 h. The plate was then washed with slow-running water, tapped on a paper towel to remove excess water, and allowed to air dry at room temperature. Subsequently, the cells were stained with an SRB solution for 30 min and washed three times with 200 µL of 1% (*v*/*v*) acetic acid. The protein-bound dye was dissolved in 200 µL of a 10 mM Tris-base solution, and the absorbance was measured using a microplate reader at 510 nm.

### 4.4. Colony Formation

A colony formation assay was employed to examine the effect of notopterol on IL-17-induced cell proliferation. A549 cells were plated at a density of 500 cells/well in a six-well plate and incubated at 37 °C in a 5% CO_2_ atmosphere overnight. Following co-treatment with IL-17 (25 ng/mL) and notopterol at concentrations ranging from 0 to 30 µM for 10 days, the resulting colonies were fixed with glutaraldehyde (6.0% *v*/*v*) for 20 min. Subsequently, the cells were stained with crystal violet (0.5% *w*/*v*) for 45 min. After staining, the colonies were washed three times with deionized water, and colony counting was performed using an iBrightTM CL-1500 imaging system. Clusters with a cell count exceeding 100 were considered colonies. 

### 4.5. Cell Cycle Analysis 

A549 cells were plated at a density of 5.0 × 10^4^ cells per well in a six-well plate and incubated overnight at 37 °C in a 5% CO_2_ environment. Subsequently, the cells were treated with various concentrations of notopterol (ranging from 0 to 40 µM) in the presence or absence of IL-17 (25 ng/mL) in DMEM containing 10% FBS for 24 h. Following treatment, the cells were fixed with ice-cold 70% (*v*/*v*) ethanol for 30 min. Nuclear DNA content staining was carried out by adding propidium iodide (PI) (50 µg/mL) and RNase A (25 µg/mL) in PBS, followed by incubation at 37 °C for 30 min in the dark. The cell cycle distribution of A549 cells was analyzed using a flow cytometer (Beckman Coulter DxFLEX), and data analysis was performed using CytExpert for DxFLEX 2.0 software.

### 4.6. Apoptosis Assay

Following the manufacturer’s instructions, the Annexin V-FITC/PI Apoptosis Detection Kit FITC (Elabscience Biotechnology Inc., Houston, TX, USA) was used to examine early/late apoptotic and necrotic cells. A549 cells were treated with various concentrations of notopterol (0–100 μM) for 48 h, and then, collected with trypsin and rinsed with PBS. After that, the cells were stained with 2.5 µL of propidium iodide (PI) and 2.5 µL of Annexin V-FITC at room temperature for 15 min. The stained cells were analyzed using the flow cytometer. CytExpert for DxFLEX 2.0 software was used for the data analysis.

### 4.7. Cell Migration Assay 

To examine the effect of notopterol on the IL-17-induced migration of A549 cells, a cell scratch assay was performed. A549 cells (1.0 × 10^5^ cells) were seeded in 12-well plate and cultured in DMEM containing 10% FBS until they reached 95% confluency. The cells were then starved for 6 h in a serum-free medium. Scratches were created using a sterile 200 ul pipette tip and we changed the starvation medium to remove cell debris. After that, cells were treated with IL-17 (25 ng/mL) and notopterol (0–40 µM) in DMEM with 0.5% FBS, and we took photos of cell recovery at 0, 24, and 48 h; then, we compared them with the control under a light microscope. Finally, the distance of wounds was measured using Image J (v1.410).

### 4.8. Cell Invasion Assay

The invasion assay was performed using the modified Boyden chamber method, as previously described. Briefly, polyvinylpyrrolidone-free polycarbonate filters (Millipore, Carrigtwohill, Tullagreen) with a pore size of 8 µm were coated with 50 µL of fibronectin (10 µg/mL) and Matrigel (10 µg/50 µL). A total of 1.25 × 10^5^ A549 cells were treated with various concentrations of notopterol (ranging from 0 to 40 µM) and placed into the upper chamber. The medium in the lower chamber contained 1% FBS, and IL-17 (25 ng/mL) was added as a chemoattractant. The cells were then incubated for 18 h. After incubation, the cells that had invaded the lower surface of the membrane were fixed with methanol and stained with 1% (*w*/*v*) toluidine blue. The migrated cells on the lower surface of the filter were counted.

### 4.9. Gelatin Zymography Assay

After cells were treated with IL-17 (25 ng/mL) and co-treated with notopterol (0–40 µM) for 24 h in DMEM with 0.5% serum, the culture supernatant was collected in equal amounts from the cells and separated using 10% polyacrylamide gels containing 0.1% *w*/*v* of gelatin in non-reducing conditions. After electrophoresis, gels were washed twice with 2.5% Triton X-100 for 30 min at room temperature to remove sodium dodecyl sulfate (SDS). Then, the gels were incubated at 37 °C for 18 h in activating buffer (50 mM Tris–HCl, 200 mM NaCl, 10 mM CaCl_2_, pH 7.4). Gels were stained with Coomassie Brillant Blue R (0.1% *w*/*v*) and destained in 30% methanol with 10% acetic acid. MMP-2 activity appeared as a clear band against a blue background. Digestion bands were quantitated using Image J v1.410 software.

### 4.10. Western Blot Analysis

After treating A549 cells with IL-17 (25 ng/mL) and notopterol (0–40 µM), the cells were collected through trypsinization, followed by centrifugation and washing with cold PBS. Subsequently, the cells were lysed using RIPA buffer containing a cocktail of protease inhibitors for 20 min on ice. The protein concentration in each cell lysate was determined using the Bradford method (Bio-Rad Laboratories, Des Plaines, IL, USA). The whole-cell lysate was then separated via SDS-PAGE electrophoresis and transferred to a nitrocellulose membrane via electroblotting. Following this, the membrane was blocked and incubated with primary antibodies in a one-step solution (Bio-Helix, New Taipei, Taiwan) for 2 h at room temperature. Subsequently, the membrane was incubated with a secondary antibody (diluted at 1:10,000) in a one-step solution for 2 h. Following incubation, the membrane was washed five times with PBS containing 5% tween for 5 min each, and then, stored in PBS before imaging. For protein visualization, chemiluminescence-based development techniques were employed, and images were captured using the iBrightTM CL-1500 imaging system. The quantitative expression of each protein was determined by analyzing band density using ImageJ (v1.410).

### 4.11. Statistical Analysis

One-way ANOVA with Dunnett’s test and an independent-samples *t*-test were performed using GraphPad prism 9 Statistics v.5.1.733. Data are presented as the average ± SD (n = 3). *p* < 0.05 was considered to indicate a statistically significant difference.

## Data Availability

Not applicable.

## Abbreviations

NSCLC	Non-small-cell lung cancer
IL-17	Interleukin-17
STAT3	Signal transducer and activator of transcription 3
MAPKs	Mitogen-activated protein kinases
Akt	AKT serine/threonine kinase 1
NF-κB	Nuclear factor kappa B
EMT	Epithelial–mesenchymal transition
Ki-67	Antigen Kiel 67
MCM2	Mini-chromosome maintenance protein 2
PCNA	Proliferating cell nuclear antigen
CDKs	Cyclin-dependent kinases
ERK	Extracellular regulated kinase
p38	p38 mitogen-activated protein kinases
JNK	c-Jun N-terminal kinase
AP-1	Activator protein 1
ECM	Extracellular matrix
